# Bilateral central retinal vein occlusion as an initial presentation of Waldenström macroglobulinemia: a case report

**DOI:** 10.1186/s13256-023-03778-4

**Published:** 2023-02-20

**Authors:** Suraj Shrestha, Elisha Poddar, Bibhav Bashyal, Aayush Adhikari, Prabin Pathak, Suman Acharya, Surendra Sapkota, Anjan Bhattarai, Samriddha Raj Pant, Anjan Shrestha

**Affiliations:** 1 Maharajgunj Medical Campus, Institute of Medicine, Kathmandu, Nepal; 2 Department of Internal Medicine, Tribhuvan University Teaching Hospital, Kathmandu, Nepal; 3 Department of Pathology, Tribhuvan University Teaching Hospital, Kathmandu, Nepal; 4 Department of Internal Medicine, Saint Agnes Hospital, Maryland, USA

**Keywords:** CRVO, Hyperviscosity syndrome, Lymphoplasmacytic lymphoma, Waldenström macroglobulinemia

## Abstract

**Background:**

Waldenström macroglobulinemia is a rare hematological malignancy and is the most common diagnosis in patients with hyperviscosity syndrome. Bilateral central retinal vein occlusion as an initial presentation of hyperviscosity syndrome in Waldenström macroglobulinemia is rare.

**Case presentation:**

A 42-year-old Nepalese male presented with sudden-onset bilateral painless blurring of vision. Fundus examination revealed bilateral, diffusely dilated, tortuous retinal veins and intraretinal deep blot hemorrhages in all four quadrants of the retina in both eyes; features of bilateral central retinal vein occlusion. Serum electrophoresis showed hypoalbuminemia with an immunoglobulin M kappa monoclonal spike. Bone marrow picture and immunohistochemistry analysis were suggestive of lymphoplasmacytic lymphoma. The patient received systemic therapy for Waldenström macroglobulinemia, along with intravitreal bevacizumab.

**Conclusion:**

Adequate hydration, plasmapheresis, and a combination of bortezomib, dexamethasone, and rituximab regimen as a systemic therapy may represent an ideal choice for patients with hyperviscosity in Waldenström macroglobulinemia.

## Background

Waldenström macroglobulinemia (WM) represents a rare hematological malignancy with an incidence of three to four cases per million people per year with a predilection for white males. The characteristic feature of WM is an uncontrolled accumulation of immunoglobulin M (IgM)-secreting malignant lymphoplasmacytic lymphoma cells in the bone marrow and other organs [1, 2].

WM is the most frequent diagnosis among patients with hyperviscosity syndrome (HVS), accounting for nearly 85% of cases of HVS [3]. However, elevated serum IgM levels can cause symptomatic hyperviscosity in only about 10–15% of patients with WM [4].

HVS is considered an oncologic emergency and can present with various manifestations. The overall incidence of bilateral simultaneous central retinal vein occlusion (CRVO) is low, with an incidence of less than 1% [5]. In addition, bilateral CRVO is also a rare presentation of lymphoproliferative diseases, including WM [6]. Only a handful of case reports are available in the literature. Here, we report a case of a 42-year-old male with bilateral CRVO as an initial presentation of WM.

## Case presentation

A 42-year-old Nepalese male, with an unremarkable medical and family history, presented with a complaint of sudden-onset bilateral painless blurred vision for 5 days. The blurring of vision was present in all eye fields, including near and far vision without redness, lacrimation, or double vision. There was no prior history of floaters in the visual field or the appearance of shade/curtain. Furthermore, the patient had no difficulty walking and speaking, and no headache, dizziness or loss of consciousness. In the past, there have been no such episodes or any symptoms necessary to seek any medical attention.

**Fig. 1:**
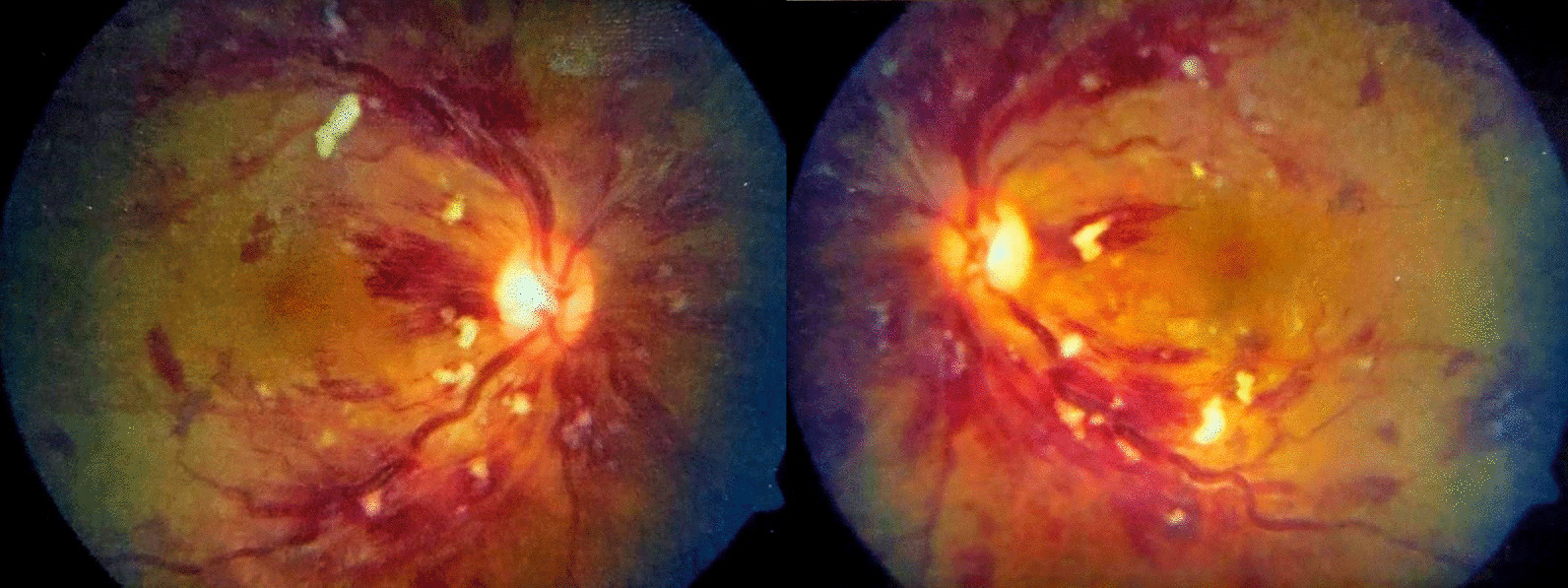
Fundus photo showing tortuous superior and inferior retinal veins with multiple areas of hemorrhage in bilateral eyes without papilledema

**Fig. 2:**
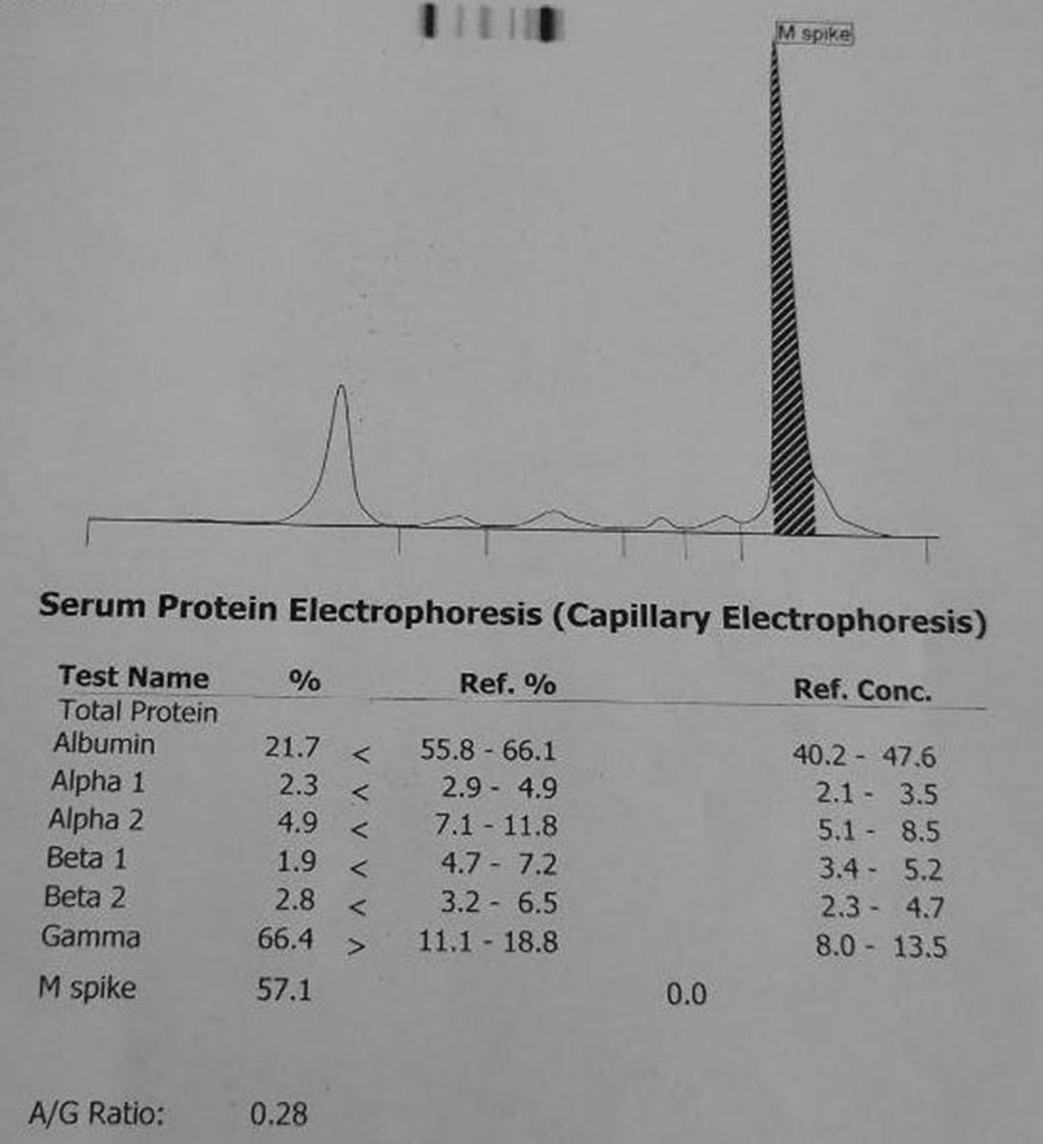
Serum protein electrophoresis showing monoclonal M-spike (before initiation of systemic therapy)

**Fig. 3:**
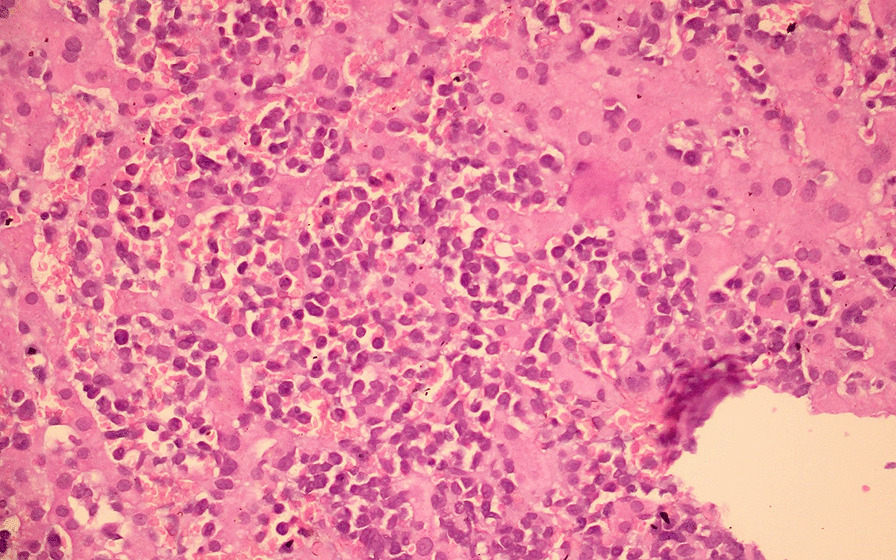
Bone marrow biopsy [hemotoxylin and eosin (H&E) stain] showing hypercellular bone marrow with lymphoplasmacytic cells

**Fig. 4:**
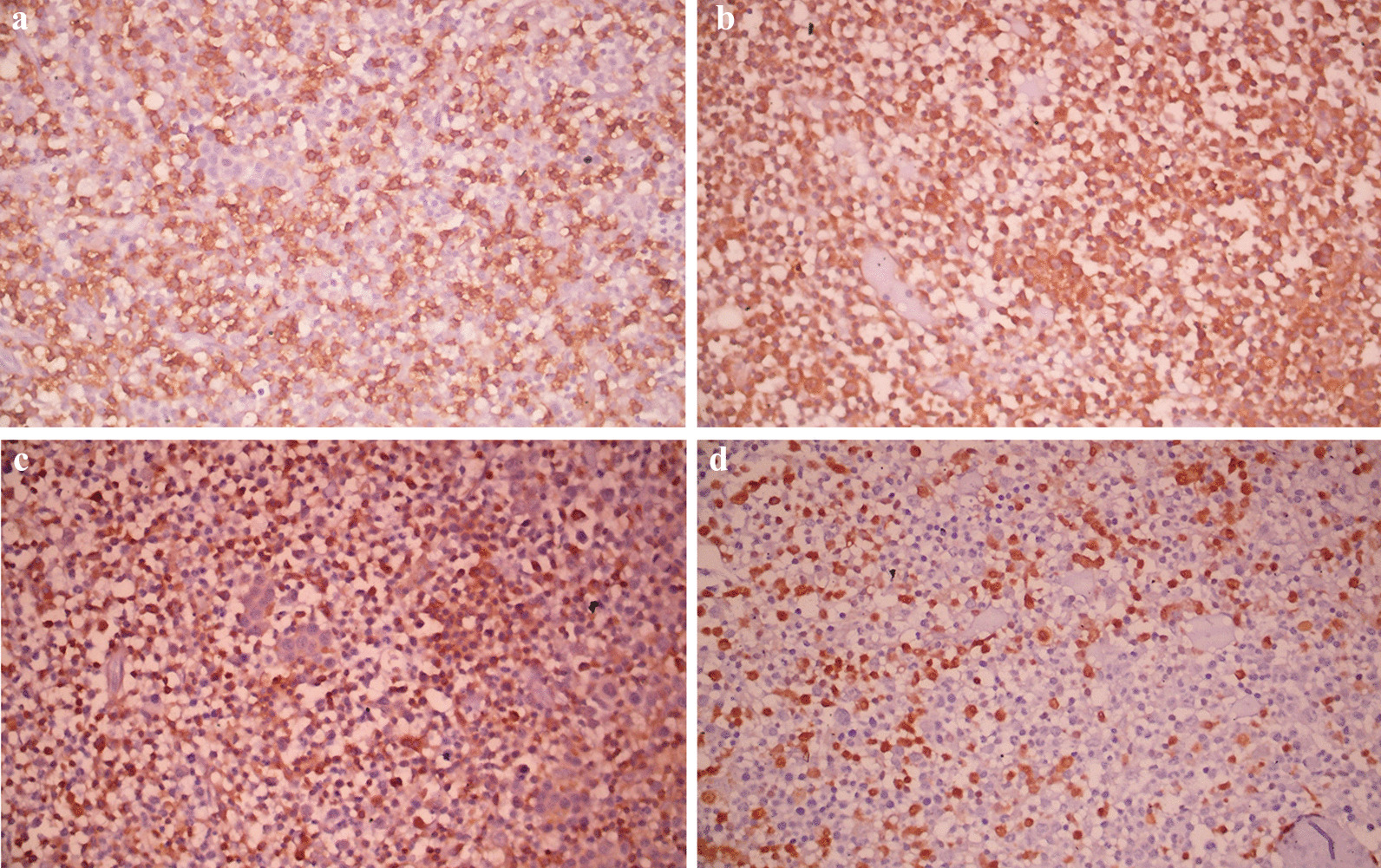
Immunohistochemistry analysis showing tumor cells positive for *CD20* (**a**), *CD5* (**b**), *BCL2* (**c**), and *MUM1* (**d**)

**Fig. 5:**
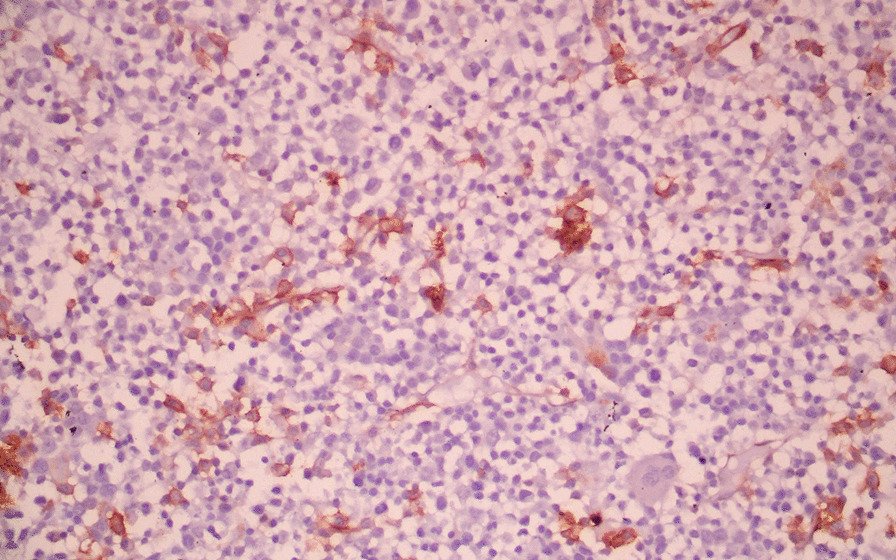
Immunohistochemistry analysis showing *CD138* positivity in plasmacytoid cells

The systemic examination of the patient, including the neurological examination, was unremarkable except for pallor. Visual acuity was 6/60 bilaterally. The anterior segment of the bilateral eyes was normal. Fundus examination revealed bilateral, diffusely dilated tortuous retinal veins, and deep-blot intraretinal hemorrhages in all four quadrants of the retina in both eyes. An optical coherence tomography (OCT) showed significant central macular edema in both eyes, more prominent in the left eye. The clinical picture was consistent with simultaneous bilateral perfused CRVO and cystoid macular edema (CME) (Fig. 1). The patient declined plasmapheresis due to financial constraints and was treated with aggressive hydration and intravitreal bevacizumab. With suspicion of hyperviscosity/thromboembolic phenomenon, further investigations were performed. Laboratory investigation showed persistent bicytopenia with normal white blood cell counts, and other laboratory parameters including blood glucose and CRP. Viral serologies [human immunodeficiency virus (HIV), hepatitis B virus (HBV), hepatitis C virus (HCV), cytomegalovirus (CMV), and Epstein–Barr virus (EBV)] and Mantoux test were negative. Antinuclear, antiphospholipid, and antineutrophilic cytoplasmic antibodies, and cryoglobulins, were negative. The Doppler study of bilateral carotid and vertebral arteries was normal. Serum electrophoresis showed hypoalbuminemia with an IgM kappa monoclonal spike (IgM percentage 56% corresponding to 29.6 g/L) in the gamma region (Fig. 2). Bone marrow biopsy showed hypercellular marrow showing an increase in lymphoplasmacytic cells, features suspicious of lymphoplasmacytic lymphoma. On immunohistochemistry analysis, the tumor cells were positive for *BCL2, CD20, MUM1, CD5,* and *CD138* in plasmacytoid cells and negative for *CD10, CD23,* and *Cyclin D1* with Ki67 of 4%. (Figs. 3, 4 and 5).

**Fig. 6:**
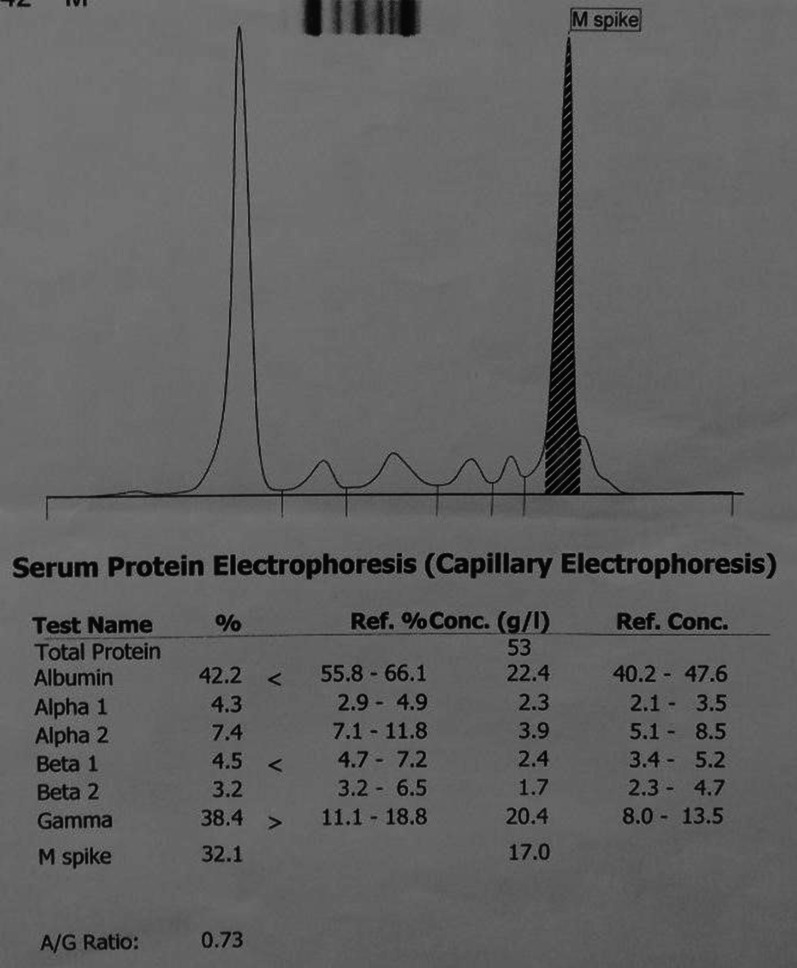
Serum protein electrophoresis showing a decrease in monoclonal M-spike (after completion of four VDR regimens)

The computed tomography (CT) scan of the chest and abdomen showed splenomegaly with no evidence of lymphadenopathy. The patient received multiple episodes of blood transfusion during the hospital stay and was started on a bortezomib, dexamethasone, and rituximab (VDR) regimen for WM. After two doses of intravitreal bevacizumab and systemic therapy for WM, the vision of the patient improved to 6/18. He received a total of six VDR regimens following which his vision has improved bilaterally significantly to 6/9. The bone marrow biopsy after the completion of chemotherapy revealed hypercellular marrow with no evidence of lymphoplasmacytic lymphoma. Moreover, the M-spike has decreased to 34% (17 g/L) in serum electrophoresis (Fig. 6). The patient is currently receiving maintenance rituximab every 2 months and is under regular follow-up.

## Discussion and conclusions

Bone marrow involvement is always present in WM, and causes varying degrees of cytopenias, depending on the degree of infiltration. The most common symptoms of WM are those caused by normocytic anemia as leukopenia and thrombocytopenia are less frequent [7]. The risk of having symptomatic hyperviscosity in WM is greater among patients with *CXCR4* mutations leading to elevated serum IgM levels [8]. This pathological increase in serum protein is responsible for various symptoms of HVS.

In general, the symptoms due to HVS are often classified into (1) general symptoms, such as fatigue, weight loss, and anorexia; (2) neurologic symptoms, such as headaches, nausea, vertigo, dizziness, ataxia, paresthesia, decreased hearing, and, rarely, coma; and (3) vascular disturbances, such as epistaxis, gingival and gastrointestinal hemorrhages or menorrhagia, congestive heart failure, retinopathy (including retinal hemorrhages), papilledema, dilated retinal veins and visual disturbances, and perfusion-related renal problems. Hyperviscosity syndrome presents with a classic triad of neurological deficits, vision loss, and mucosal bleeding [9]. Our patient had a bilateral simultaneous decreased vision, and splenomegaly.

HVS-related retinopathy with central retinal hemorrhages and vascular dilation is seen in patients with WM. Venous stasis retinopathy with subsequent hypoxia of retinal vascular endothelial cells and breakdown of the blood–retinal barrier, and intraretinal IgM infiltrating the subretinal area through focal defects of the outer retinal layers is the most dominant theory [10]. In addition, vascular tortuosity, retinal hemorrhage, exudates, and retinal vein occlusion due to hypoxia of retinal vascular endothelial cells have been considered the cause of vision loss [11]. Also, abnormal platelet aggregation and adhesiveness due to nonspecific IgM coating in WM promote the formation of venous thrombosis leading to CRVO [12]. All these microvascular changes are responsible for visual derangements such as blurred vision or double vision [3].

“Sausage link” or “boxcar” engorgement of retinal veins along with papilledema, flame-shaped hemorrhages, or exudates are the classical findings of CRVO that allow ophthalmologists to make a prompt diagnosis and treatment in an appropriate clinical setting. In addition, the finding of bilateral optic nerve edema, along with vascular congestion and tortuosity, warrants the search for underlying hyperviscosity syndrome including WM [3]. The finding of simultaneous bilateral CRVO prompted further investigations that eventually led to the diagnosis of WM in our case.

Plasma exchange/plasmapheresis and chemotherapy are the mainstay of the treatment of HVS in WM. Plasmapheresis has been known to reduce serum viscosity and hyperviscosity-related retinopathy significantly. It can even reverse associated retinopathy and prevent other complications (for example, cerebral stroke) if HVS is identified in a timely manner. In the largest case series with three cases of bilateral CRVO in WM, all patients were treated with plasmapheresis and systemic immunosuppressive therapy (different combinations of cyclophosphamide, fludarabine, chlorambucil, and rituximab), two of whom had better visual acuity after treatment while the other one was unchanged [13]. Reduction of IgM by ~ 50% with plasmapheresis patients with HVS has been shown to improve retinopathy in all patients [14]. Furthermore, since dehydration can worsen HVS and patients are often dehydrated, careful fluid administration is advised [3]. However, some patients may ultimately suffer from decreased vision due to ischemic maculopathy, despite an improvement in the clinical appearance of the retina. This has been attributed to capillary occlusion of the perifoveal area and retinal pigmentary alterations, as well as mechanical obstruction of the capillary bed by aggregated blood components or a true thrombus that was not reversed by plasmapheresis [15, 16].

Plasmapheresis is beneficial in hyperviscosity syndrome; however, it does not affect the underlying disease and concomitant chemotherapy has to be initiated often. As no cure is available for WM, highly variable regimens are currently in use and no single treatment has been standardized for symptomatic WM. The current treatment of patients with WM depends mainly on alkylating agents, proteasome inhibitors, *BTK* inhibitors, and *anti-CD20* monoclonal antibodies. Rituximab, a chimeric *anti-CD20* monoclonal antibody, is the most common agent used, alone or in combination, in the treatment of WM. However, rituximab-based therapies take months for the best response and would not be considered first-line if the primary objective of therapy is rapid reduction of the viscosity level [17, 18]. In such cases, bortezomib-based therapies produce very rapid responses in 85% of patients, with a flare in only 11% [19]. The combination of bortezomib, dexamethasone, and rituximab (VDR) as a systemic therapy may represent an ideal choice for patients with hyperviscosity in whom rapid reduction of paraprotein is needed [20]. In our patient, early recognition and timely management of hyperviscosity syndrome with adequate hydration, and systemic therapy with the VDR regimen helped achieve a significant improvement in vision and marrow picture of WM.

Retinal hyperviscosity, which is usually bilateral, is often related to dysproteinemia, such as Waldenström macroglobulinemia or multiple myeloma. Therefore, in any patient with simultaneous bilateral CRVO, early work-up including serum protein electrophoresis should be performed. Urgent hematological consultation should be sought, as timely diagnosis and initiation of systemic therapy can improve visual acuity.

## Data Availability

All the required information is available in the manuscript.

## Abbreviations

CRVO: Central retinal vein occlusion
CRP: C-reactive protein
HVS: Hyperviscosity syndrome
WM: Waldenström macroglobulinemia
VDR: Bortezomib, dexamethasone, rituximab
